# 

**Published:** 1923-07

**Authors:** 


### Contents

Event and Comment .......... 289
The Dental Convention and Men We Meet There .......... 290
Dental College Affiliates with Cincinnati University .......... 301
General Statement in Regard to the Forsyth Dental Infirmary and Its Purposes .......... 303
Chicago Dental Society .......... 304
Radiant Light and Heat .......... 305
The Endocrine Glands in Relation to Dental Nutrition and Caries .......... 312

